# The Community Health Workers and Mobile Health for Emerging Adults Transitioning Sickle Cell Disease Care (COMETS) Trial: Protocol for a Randomized Controlled Trial

**DOI:** 10.2196/69239

**Published:** 2025-09-04

**Authors:** Tanisha D Belton, Caren M Steinway, Olivia Teng, Justine Shults, Lamia P Barakat, Banu Aygun, Abena Appiah-Kubi, Lori E Crosby, Omar Niss, Biree Andemariam, Lisa A Schwartz, Samantha Luma, Kyle A Smith, Tracey B Johnson, David M Rubin, Kim M Smith-Whitley, Sophia Jan

**Affiliations:** 1 PolicyLab Division of General Pediatrics Children's Hospital of Philadelphia Philadelphia, PA United States; 2 Department of Pediatrics Northwell Health New Hyde Park, NY United States; 3 Department of Pediatrics Donald & Barbara Zucker School of Medicine at Hofstra/Northwell Hempstead, NY United States; 4 Department of Pediatrics Children's Hospital of Philadelphia Philadelphia, PA United States; 5 Department of Biostatistics, Epidemiology, and Informatics Perelman School of Medicine at the University of Pennsylvania Philadelphia, PA United States; 6 Division of Oncology Children's Hospital of Philadelphia Philadelphia, PA United States; 7 Pediatric Hematology Oncology and Stem Cell Transplantation Cohen Children's Medical Center New Hyde Park, NY United States; 8 Behavioral Medicine and Clinical Psychology Cincinnati Children's Hospital Medical Center Cincinnati, OH United States; 9 Department of Pediatrics Cincinnati Children's Hospital Medical Center Cincinnati, OH United States; 10 Division of Hematology Cincinnati Children's Hospital Medical Center Cincinnati, OH United States; 11 New England Sickle Cell Institute University of Connecticut Health Farmington, CT United States; 12 Division of General Pediatrics Children's Hospital of Philadelphia Philadelphia, PA United States

**Keywords:** sickle cell disease, pediatrics, health care transition, adolescents and young adults, self-management, quality of life, mobile health, mHealth, community health worker, mobile phone

## Abstract

**Background:**

Transitioning from pediatric to adult sickle cell disease (SCD) care is challenging for emerging adults (aged 17-25 years). This period is marked by a 7-fold increase in mortality rates and has the highest rates of hospitalizations, emergency room visits, and hospital readmissions compared with children living with SCD. These challenges are exacerbated by fragmented care coordination, difficulty navigating adult health care systems, and increased self-management responsibilities.

**Objective:**

This study aims to compare the effectiveness of 2 interventions designed to support emerging adults living with SCD during this transition: a mobile health (mHealth) app and community health worker (CHW) support to standard care.

**Methods:**

The Community Health Workers and Mobile Health for Emerging Adults Transitioning Sickle Cell Disease Care (COMETS) trial is an ongoing multicenter, 3-arm, open-label randomized controlled trial; 375 emerging adults (aged 17-25 years) are being enrolled and randomized 1:1:1 to (1) a 6-month CHW intervention focused on self-management skills, symptom tracking, care coordination, and transition planning; (2) a 6-month mHealth self-management program (enhanced iManage application) with tailored SMS text messaging (THRIVE [Texting Health-Related Resources to Inform, Motivate, and Engage] 2.0); or (3) enhanced usual care (control). Participants are followed for 18 months. The primary outcome is the change in self-reported health-related quality of life assessed using the PedsQL SCD module. Secondary outcomes include acute care use (hospitalizations and emergency department visits), patient activation, self-management behavior, and successful transfer to adult hematology care.

**Results:**

The institutional review board at Children’s Hospital of Philadelphia approved this study in June 2018. Recruitment began in January 2019 and ended in December 2022; we completed data collection in November 2024. We have enrolled a total of 405 participants.

**Conclusions:**

This trial addresses a critical gap in transition intervention research for young adults with SCD. It will provide evidence on the comparative effectiveness of 2 promising interventions (CHW and mHealth) and inform the development of scalable and sustainable transition support programs. Findings will have implications for improving health-related quality of life, reducing acute care use, and promoting successful transition to adult-centered SCD care for this vulnerable population.

**Trial Registration:**

ClinicalTrials.gov NCT03648710; https://clinicaltrials.gov/study/NCT03648710

**International Registered Report Identifier (IRRID):**

DERR1-10.2196/69239

## Introduction

### Background

Emerging adults with sickle cell disease (SCD) face a 7-fold increase in mortality rates during the transition from pediatric care to adult care [1]. In addition, this subpopulation (aged 17-25 years), experiences the highest rates of hospitalizations, emergency room visits, and hospital readmissions compared with children living with SCD [2]. This alarming increase in mortality and acute care use stems partly from the cumulative effects of SCD leading to organ dysfunction, with the risk of comorbidities such as stroke and silent infarcts worsening as individuals age [3,4]. In addition, during this transition process, emerging adults with SCD experience fragmented care coordination, which increases their vulnerability.

Emerging adults with SCD face shifts in insurance, inconsistent transfer processes, and a lack of comprehensive adult-focused programs [4-6]. For instance, while adherence to chronic transfusion therapy and hydroxyurea therapy can substantially mitigate the risk of neurological complications and other adverse outcomes, inadequate care coordination during the transition to adult care, coupled with suboptimal patient engagement and SCD self-management skills, markedly heighten the risk of neurological complications [6-9]. These complications can exert enduring and profound impacts on physical function, educational attainment, employment, income, and overall health-related quality of life (HRQOL).

Beyond health system challenges, emerging adults face additional psychosocial challenges, including the struggle for financial independence and insurability, which can be complicated by job instability and the loss of dependent coverage on parents’ insurance plans [10]. Transportation barriers may also limit access to necessary medical appointments or therapies, especially for those living in areas with limited adult health care resources [8]. In addition, many emerging adults with SCD experience limited access to mental health care, which can exacerbate issues such as depression, anxiety, and the psychological burden of managing a chronic illness [11]. Social determinants of health, including poverty, housing instability, food insecurity, and lack of educational opportunities, also further complicate the ability of emerging adults with SCD to manage their condition effectively and navigate the health care system [11]. Furthermore, the absence of adequate social support and educational resources compounds these challenges, leaving emerging adults with SCD underprepared and less equipped to navigate their transition effectively [6,12].

Disruption in continuity of care, combined with socioeconomic factors, can lead to worse health outcomes, and an increased risk of complications show the need for effective interventions [11,12]. Community health worker (CHW) programs and mobile health (mHealth) platforms have shown promise in addressing these challenges and improving health outcomes for chronic disease transition patients [13,14]. Research shows that CHW programs enhance chronic disease management and health care navigation, improving outcomes in low-resource settings for conditions such as asthma, hypertension, and diabetes [15,16]. For instance, the IMPaCT CHW program demonstrated that participants in the intervention arm received more timely posthospital care and better discharge communication and experienced fewer readmissions [13].

Similarly, mHealth platforms have been effective in promoting self-management and adherence among both adults [17,18] and emerging adults with chronic conditions, including diabetes and post–liver transplant care [14,19,20]. A randomized controlled trial (RCT) of MD2Me, an internet and mobile phone–delivered disease management intervention using automated SMS text messaging algorithms, showed that emerging adults with type 1 diabetes, inflammatory bowel disease, and cystic fibrosis in the intervention arm significantly improved disease self-management and health-related self-efficacy [14]. This technology holds great promise for adolescents with chronic diseases due to the widespread use of smartphones [21]. Integrating CHW and mHealth initiatives can substantially alleviate health care transition challenges and address psychosocial factors, thus improving care continuity and outcomes for patients with chronic diseases.

### Objective

Gaps in evidence highlighted in the study by Viola et al [6] demonstrate the need for multisite RCTs focusing on long-term care outcomes. Current research, while promising, offers inconclusive results due to limited sample sizes and methodological constraints. Recognizing these limitations, our study aims to bridge this gap by conducting comprehensive multisite RCTs that evaluate both clinical and psychosocial outcomes, ultimately aiming to enhance chronic disease management and overall health during this critical period. We hypothesize that emerging adults in the CHW or mHealth arm will have increased HRQOL compared with those in the enhanced usual care arm.

## Methods

### Theoretical Framework

Our intervention leverages 2 key frameworks: the social-ecological model for adolescents and young adults readiness to transition (SMART) model and the chronic care model. Effective transition care, as emphasized by the SMART model, requires integrating both preexisting and modifiable factors among patients, parents, and health care providers that influence transition readiness. Modifiable factors, such as knowledge, self-management skills, confidence (or self-efficacy), and social support, are critical targets for enhancing transition readiness, especially for individuals managing chronic conditions. The SMART model has been validated across various populations and has been updated for health equity after this trial was designed, including childhood cancer survivors and their parents and health care providers [11,22,23].

For individuals with SCD, as well as their parents and health care providers, the ability to independently manage their SCD is recognized as a crucial goal during the transition to adult care [6,24-26]. Self-management behaviors, defined as the daily, self-motivated, and collaborative activities required to manage symptoms, are essential for successful disease management. In the context of SCD, these behaviors include self-awareness; seeking emotional support; managing diet and hydration; avoiding alcohol, tobacco, and drugs; and adhering to prescribed treatments such as daily hydroxyurea or regular transfusion and chelation therapy.

**Figure 1:**
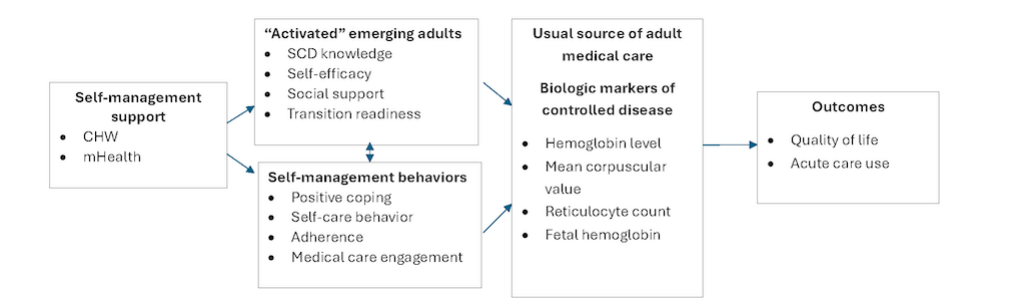
Key clinical question and conceptual model. CHW: community health worker; mHealth: mobile health; SCD: sickle cell disease.

Self-management support is a foundational element of the chronic care model [27], which is widely regarded as an effective framework for improving chronic illness care. According to this model, improved health outcomes, including better disease control, a smoother transition to adult care, and enhanced quality of life (QOL), are achieved through the activation and engagement of patients in self-management behaviors, supported by proactive health care teams. In this context, individuals who are more “activated” tend to exhibit higher levels of the modifiable factors outlined by the SMART model, such as increased knowledge, self-efficacy, and social support, all of which are crucial for successful self-care and transition. We chose to target these modifiable factors because patients with SCD and their parents and clinicians agree that it is critical to engage in disease self-management to optimize health during the transition period (Figure 1) [28].

### Study Design

The Community Health Workers and Mobile Health for Emerging Adults Transitioning Sickle Cell Disease Care (COMETS) study is an ongoing pragmatic, multicenter, 3-arm, open-label RCT. A total of 5 pediatric SCD centers located in 4 states (Connecticut, New York, Ohio, and Pennsylvania) are participating in this study. Eligible participants are randomized 1:1:1 across sites to a 6-month CHW program, 6-month mHealth self-management program with tailored SMS text messaging, or to enhanced usual care (control).

### Specific Aims

The overall goal of this study aims to answer the question “How can community health workers and mobile applications help me to improve my ability to take care of myself (activated patient, self-management), stay connected with my doctors (usual source of care), avoid visits to the ER and hospital (acute care use), and ultimately maximize my quality of life while I am transitioning from my pediatric to adult doctors?”

#### Aim 1

Compare the effectiveness of 2 self-management support interventions versus enhanced usual care to improve HRQOL and acute care use for transitioning youth with SCD. This aim will test the following hypothesis: Emerging adults in the CHW arm or mHealth arm will have higher quality of care and lower acute care use than those in the usual care arm at 6, 12, and 18 months.

#### Aim 2

Identify and quantify whether patient activation, self-management behaviors, biologic markers, and transfer to adult care are mediators of intervention treatment effects. This aim will test the following hypotheses:

- Patients in the CHW arm and mHealth arm will have greater patient activation and self-management behaviors compared with those in the usual care as 6 months.
- Patients with greater patient activation and self-management behaviors will have better treatment outcomes compared with those in usual care at 6, 12, and 18 months.
- Patients in the CHW arm and mHealth arm will affect the total number of adult hematology visits and magnitude of biologic markers compared with those in usual care at 18 months.
- Patients with greater adult hematology visits or higher biologic markers will have better outcomes compared with those in usual care at 18 months.

#### Aim 3

Identify individual and family factors that moderate intervention treatment effects. We will test the following hypotheses:

- Emerging adults with SCD and high disease severity in the CHW arm or mHealth arm will have higher HRQOL and lower QOL or lower acute care use than those in the usual care arm at 6, 12, and 18 months.
- Older adults (aged ≥22 years) with SCD in the CHW arm or mHealth arm will have higher QOL or lower acute care use than those in the usual care arm at 6, 12, and 18 months.
- We will also explore whether patient demographics (gender, education or employment, health literacy, insurance type, and zip code), family demographics (living with parent or caregiver and maternal education), and recruitment method moderate outcomes in the CHW arm or mHealth arm compared with similar patients in the usual care.

#### Exploratory Aim

Explore the association of enhancements to usual care on pediatric and adult care use. We will test the hypothesis that enhancements to usual care will decrease pediatric acute use and increase adult acute use.

### Study Population, Recruitment, and Enrollment

Eligible participants are young adults aged 17 to 25 years with SCD diagnosis of all genotypes who plan to transition to an adult hematologist within 12 months; have access to a mobile device, tablet, or computer; and can interact with a mobile- or web-based program. Eligible participants are identified by reviewing each recruitment site’s daily hospitalized patients, weekly hematology appointments, and hematology practice registries. Across the 5 pediatric SCD centers, we estimate that there will be 745 eligible patients over the recruitment period, with an equal distribution between male and female patients.

**Figure 2:**
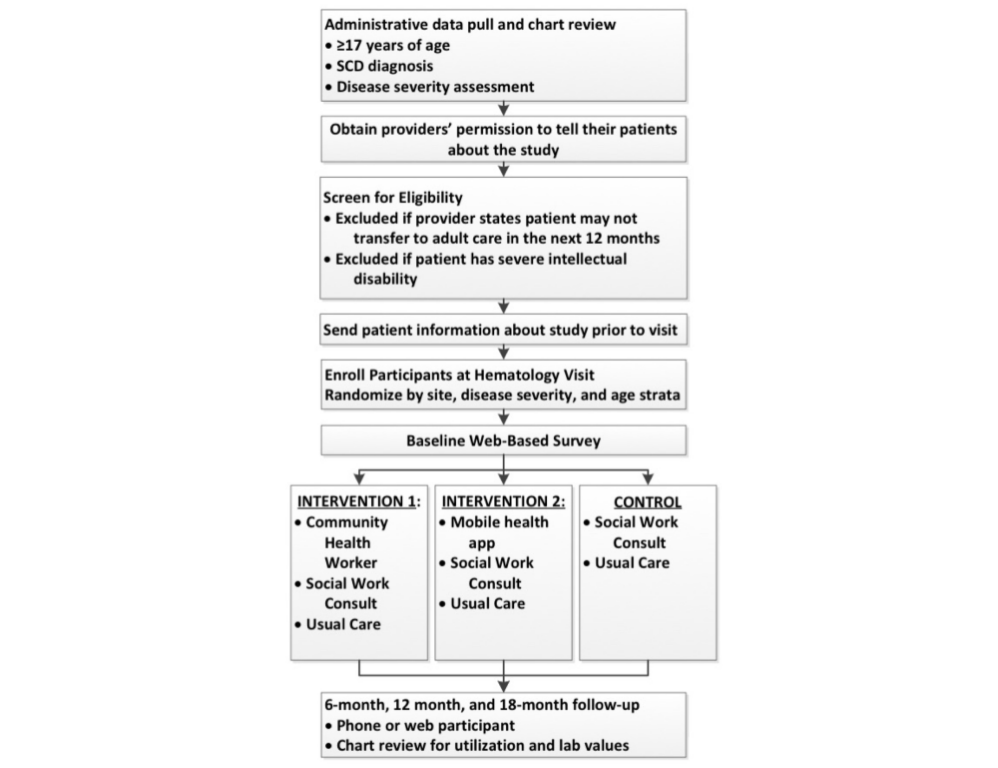
Community Health Workers and Mobile Health for Emerging Adults Transitioning Sickle Cell Disease Care (COMETS) study flowchart among emerging adults with sickle cell disease (SCD).

Recruitment strategies include in-person enrollment during appointments or admissions and mailed or emailed communications with a recruitment letter, study flyer, and a recruitment video. During the COVID-19 pandemic, telephone recruitment and electronic consent were added to maintain recruitment goals. Retention strategies include completion of enrollment and follow-up procedures in-person, telephone, or an email or text, automatic reminders, and incentives for effort. Figure 2 provides an overview of the study screening and enrollment process.

### Ethical Considerations

#### Institutional Review Board Approval and Consent

The COMETS study was funded in September 2017. Institutional review board (IRB) approval was received in June 2018 (IRB 18-015106) at the prime institution, the Children’s Hospital of Philadelphia (CHOP), and submitted to each participating site for site-level approach. This study was registered in August 2018 (NCT03648710). The study is being conducted in accordance with the IRB-approved protocol (December 15, 2020).

Informed consent was obtained from all study participants before their involvement in the research by a study coordinator. Participants were provided with comprehensive information about the study’s objectives, procedures, potential risks, and their rights, including the option to withdraw at any time without consequence. Multiple enrollment options were provided, including in-person and electronic consent via REDCap (Research Electronic Data Capture; Vanderbilt University). Participants who were aged 17 years at the time of enrollment were reconsented at the age of 18 years at an upcoming appointment, via phone or via REDCap using an electronic consent. This study was granted a waiver of parental permission due to the minimal risks associated. A waiver of documentation of consent was also granted for those aged 17 years at the time of enrollment, as they were reconsented once they turned 18 years during the study period.

#### Privacy and Confidentiality

All survey data were entered and stored in REDCap. Data linkage was conducted at the CHOP via staff from the Data and Biostatistics Data Unit. Linkage used movement reference number, first or last name, date of birth, and gender, where appropriate. To ensure confidentiality of patient information, the dataset was deidentified after a master file of linked data was created.

#### Compensation

Participants received compensation for completing surveys at designated time points: US $50 (baseline), US $75 (6 months), US $100 (12 months), and US $150 (18 months). Participants randomized to the mHealth app arm could earn up to US $3 per month for engagement, with incremental rewards for consecutive usage streaks. Additional incentives of US $5 and US $11 are provided for sustained engagement at 3 and 6 months. Payments are managed through ClinCards, a secure, reloadable debit card. Compensation amounts were determined in consultation with patient and parent coinvestigators.

#### Risk and Safety Monitoring

The study is considered minimal risk, with primary concerns including breach of confidentiality and participant discomfort with study measures. These risks were mitigated through data deidentification, password-protected storage, and voluntary participation.

For both intervention arms, there is a risk that participants may communicate urgent issues to be handled by a health care provider. To mitigate this risk, participants were provided with direct instruction at the time of consent and randomization, and constant monitoring of peer-to-peer communication in the mHealth app by study staff. Study staff who received communication from participants routed the information to the study site social worker, where appropriate.

The principal investigator was responsible for monitoring the safety of study participants and complying with all reporting requirements. Any adverse events were reported immediately to the CHOP IRB.

### Randomization

Random assignment takes place after enrollment and the completion of the baseline survey. A document including stratification and randomly permutated blocks of unequal sizes (to prevent providers and patients from manipulating the randomization) was used in REDCap’s randomization module. Enrolled participants will be randomly assigned to a treatment arm after being stratified by recruitment site, age (≤21 y and ≥22 y), and disease severity (moderate and severe), resulting in 12 different randomization strata [29,30]. Severe SCD disease will be determined by chart review and defined by the following: (1) any history of stroke, acute chest syndrome, or ≥3 emergency room visits within the 3 years before enrollment, which are all indications for disease modifying therapy, such as daily hydroxyurea or monthly exchange transfusion therapy [31-33]; and (2) receipt of monthly exchange transfusion therapy, defined by ≥2 exchange transfusions in an outpatient setting within 6-month period [33]. Severe disease will be defined as any history of acute chest syndrome, stroke, or ≥3 hospitalizations in the past 3 years [31,32].

This is a nonblinded study because the nature of the intervention prevents patients from being blinded to their study arm.

### Control Arm: Enhanced Usual Care

Enhanced usual care, standardized across all sites through transition or transfer of care checklist, is administered by the social work team at each recruitment site. During the intervention development phase, all recruitment site investigators reviewed and decided on the elements to be included in this checklist based on transition elements at their institution. The checklist is reviewed at the 12-month time point in the trial with the participant during a scheduled hematology visit. Elements of the checklist include (1) patient seen by the pediatric provider, with the parent outside the examination room at least once, (2) a social work consult to screen and address sociodemographic risk factors, (3) information on health insurance availability provided to patient, (4) adult hematologist identified, (5) adult primary care provider identified, (6) medical release signed, and (7) medical record viewable or sent to adult provider. Elements of the checklists are based on existing checklists by organizations such as the Transfer of Care Checklist developed by Got Transition or Center for Health Care Transition Improvement [34] and those developed by the National Institute for Child Health Quality [35]. All study participants received standardized enhanced usual care.

### Enhanced Usual Care+Peer CHW

The CHW program is modeled after the IMPaCT program developed by IMPaCT Care, Inc [13,36,37]. Components of the CHW program include (1) development of patient-centered goals and individualized action plan around self-care, symptom tracking, and transition to adult care; (2) provision of information, skills, and tips; and (3) tailored peer support using telephone calls or visits.

To identify the components of the CHW intervention, web-based work group meetings were held to define the role and training components [38]. Training topics included local hospital and community knowledge, SCD and transition to adulthood education, basic clinical research training, foundational CHW skills and concepts, and trial-specific aims and resources.

Training components included IMPACT’s 2-week training course, onboarding and orientation at their respective sites, and Collaborative Institutional Training Initiative for clinical research. With an emphasis on ongoing support and education, CHWs meet weekly with a trial supervisor and their site supervisor to review caseloads and address any concerns. Monthly group huddles are held to support peer-to-peer learning for both CHWs and their supervisors. CHWs are expected to contact participants weekly during the 6-month intervention period to remain consistent with other successful protocols [39,40].

### Enhanced Usual Care+mHealth

Participants randomized to the mHealth arm will download an enhanced version of iManage [41] called iManage SCD, developed by Crosby et al in collaboration with adolescents and young adult patients with SCD [28,38]. Key components of iManage SCD include (1) development of patient-centered goals around self-care, symptom tracking, and the transition to adult care; (2) provision of information, skills, and tips to support these goals; (3) virtual peer support, where users can encourage others, form teams, and interact with fellow youth with SCD; and (4) daily symptom tracking and visual monitoring of goal completion. iManage SCD is an mHealth application accessible via smartphone, tablet, or web and has been shown to be highly feasible and beneficial for emerging adults with SCD.

Enhancements to the iManage application were identified through the convening of a multi-stakeholder group, including an application development firm, clinicians, researchers, and young adults with SCD across the 5 identified recruitment sites. The development process of the enhanced application has been described [28]. Interviews with stakeholders explored 8 topics, including (1) social behaviors and entertainment, (2) treatment, (3) pain impact, (4) pain management, (5) SCD knowledge, (6) lifestyle and goals, (7) communication and support, and (8) envisioning the future. Final components of the application include health behavior challenges, tracking of medications and symptoms, an educational material library, discussion boards, and medical summary. User engagement was tracked through analytic tools integrated within the mHealth app [27].

In addition, daily tailored texting (THRIVE 2.0) [42], based on patient-defined goals were delivered to participants randomized to the mHealth arm of the study. Preliminary studies have found that THRIVE 2.0 improves health-promoting behaviors such as sunscreen use among adolescent and young adult cancer survivors. These SMS text messages were tailored around modifiable factors and delivered twice a week. Two types of messages were delivered, educational and interactive. Messages focused on SCD knowledge, skills and efficacy, relationships, self-management, school or work, and coping.

Participants will receive training on how to use and customize iManage SCD during enrollment and have access to a helpdesk for support. The content on iManage SCD was continuously monitored by research staff. Participants were able to access the application after the 6-month active intervention period.

Both intervention arms include the same core components, with one delivered by a CHW and the other through a digital platform. Both interventions were designed to support self-management by facilitating patient-centered goal setting, providing tailored information and skill-building, and offering peer support. In the mHealth intervention, these components are adapted to be delivered via the app, mirroring the CHW’s role in engaging participants through structured content and interactive features.

#### Data Collection

##### Overview

Participants are followed with measures related to HRQOL, acute care use, patient activation, self-management behaviors, and experiences with the COVID-19 pandemic collected through web-based survey, phone, or in-person at baseline and at 6, 12, and 18 months. Disease severity, health care use, and biologic markers will be measured from the electronic medical record (EMR) at baseline and at 6, 12, and 18 months. Study data are stored and managed using the REDCap web-based system, overseen by the CHOP. Site study coordinators are responsible for data entry at their respective sites.

##### Baseline Clinical and Demographic Information

Baseline data will be gathered from EMRs and include age, SCD genotype, history of intellectual disability, depression, anxiety, stroke, acute chest syndrome, emergency room visits, and prescribed disease-modifying therapies (hydroxyurea or exchange transfusions).

##### Intervention Adherence Process Measures

For the mHealth intervention, adherence will be tracked using app usage metrics (eg, days and frequency of access, responses to SMS text messages, and goal progress) through REDCap. CHW intervention adherence includes contact frequency (in-person, phone, email, and SMS text messaging) and goal attainment. Usual care measures include social work consults and chart review transition checklists, tracking key actions such as insurance review and provider identification.

##### HRQOL: Primary Outcome

HRQOL will be measured using the PedsQL SCD module [31,41], a validated 43-item tool that assesses pain, emotional health, treatment experience, and communication.

##### Health Care Use

Health care use data, including acute care (emergency department visits and hospital admissions) and ambulatory care, will be collected from patient reports, EMRs, and Medicaid billing data.

##### Validation of Health Care Use Data

Patient-reported data on health care use were validated with EMR data, calculating annualized acute care use and ambulatory care use for both pediatric and adult hematology.

##### Measures of Self-Management Behavior

Self-management will be measured through several tools: the Brief Coping Orientation to Problems Experienced for coping strategies [43], the Jenerette Self-Care Assessment Tool for self-care actions [44], the Medical Adherence Measure for medication adherence [45,46], medical chart reviews for appointment no-shows, and chronic transfusion adherence (based on patient report and chart review).

##### Measures of Patient Activation (Mediator)

Patient activation will be assessed using the Sickle Cell Disease Knowledge Questionnaire [47], the Transition Readiness Assessment Questionnaire [48], and the Medical Outcomes Study Social Support Survey [49].

##### Experience With the COVID-19 Pandemic

A 33-question survey will be used to assess COVID-19 symptoms, mental health impacts, trauma, social distancing effectiveness, care access issues, and telehealth satisfaction, adapted from the Johns Hopkins COVID-19 Community Response Survey [50] and US Census Household Pulse Survey [51]. These questions were chosen with input from young adult stakeholders and site principal investigators.

##### Biologic Marker of Disease Control (Mediator)

Biologic markers, including hemoglobin levels, mean corpuscular volume, reticulocyte count, and fetal hemoglobin percentage, will be obtained from chart review. These markers reflect anemia, hydroxyurea adherence, stroke risk, and disease severity (Table 1).

**Table 1:** Patient-reported scales, characteristics, and source.

Measure		Items, n	Source
**Primary outcome measures**			
	Pediatric Quality of Life Inventory sickle cell disease	43	Panepinto et al [31], 2013
**Activated emerging adult patient measures (mediators)**			
	Sickle Cell Disease Knowledge Questionnaire	23	Original scale: Armstrong et al [52], 1993, used in Barakat et al [53], 2010, Logan et al [54], 2002
	13-item Patient Activation Measure	13	Logan et al [54], 2002; Hibbard et al [55], 2005
	Transition Readiness Assessment Questionnaire	20	Sawicki et al [56], 2011; Wood et al [57], 2014
	Medical Outcomes Study Social Support Survey	19	Sherbourne and Stewart [49], 1991
**Self-management behavior measures (mediator)**			
	Brief Coping Orientation to Problems Experienced	28	Gil et al [58], 1991
	Medication Adherence Measure	17	Zelikovsky and Schast [45,46], 2008
**COVID-19 pandemic measures**			
	COVID-19 symptoms and testing experience: symptoms; diagnosis	4	Mehta et al [50], 2020
	COVID-19 and mental health impacts: mental health	10	Mehta et al [50], 2020 and Harkness et al [59], 2020
	COVID-19 and violence and trauma: violence; fear of violence	3	Mehta et al [50], 2020
	COVID-19 and social distancing: social distancing; social Impact	10	Mehta et al [50], 2020
	Reduced access to care	2	US Census Bureau [60], 2020
	Satisfaction with telehealth services	4	Parmanto et al [61], 2016

### Outcomes

The primary outcome of the COMETS study will be the change in HRQOL. The secondary outcomes are acute care use (hospitalizations and emergency department visits), patient activation, self-management behaviors, and successful transfer to adult hematology care.

### Statistical Methods

This study is ongoing; therefore, the statistical analyses have not been performed.

### Sample Size

Sample size calculations are based on our primary end point of change in HRQOL and health care use for each participant over their study participation [62]. Details of the sample size calculations are presented in Multimedia Appendix 1.

### Primary Analysis

Analyses will be conducted in Stata (version 18.0; StataCorp), with 2-sided tests of hypothesis and a *P* value <.05 as the criterion for statistical significance.

To compare the effectiveness of 2 self-management support interventions (CHWs and mHealth) versus enhanced usual care on the primary outcomes (HRQOL and acute care use), we will first compare groups at baseline using chi-square tests for categorical variables and 1-way ANOVA or Kruskall-Wallis, as appropriate, for continuous variables. We will use descriptive summaries and graphical displays to evaluate the distribution of and relationship between variables. When required, transformations to achieve normality will be considered.

The initial analysis will use 1-way ANOVA to test the hypothesis that the 3 treatment groups showed no change from the end of the intervention to the baseline in the primary and secondary outcomes. If the hypothesis of equal group means is rejected (there was a change detected), 3 post hoc tests will be applied (with Bonferroni adjustment for multiple testing) to identify pairwise differences between treatment groups. We postulate that a difference in change of 10 points in PedsQL SCD total between groups will be clinically meaningful.

Next, we will construct longitudinal models that include baseline, 6-, 12-, and 18-month measurements as outcomes. Longitudinal models account for correlation of measures within emerging adult over time; use all data collected; allow for and can be used to adjust for dropout or nonadherence to assigned treatment; can adjust for differences in patient characteristics not balanced by randomization; can include indicator variables for clinical site; and permit continuous, count, and binary outcomes. We will build models using generalized estimating equations (GEEs) or quasi-least squares (QLS) [63]; QLS is based on GEE but allows for implementation of the Markov correlation structure that is appropriate if participants intermittently miss appointments. Mixed effects models may also be considered. For example, mixed models can be used to relax the assumption of constant variance that is required for QLS and GEE; they will, therefore, be useful if residual diagnostics for GEE or QLS suggest an increase in the variance of outcomes over time.

The longitudinal models will include indicator variables for visit, group, and time-by-visit group interaction terms (if appropriate, time will be included as a continuous variable). If the time-by-group interaction terms differ significantly from 0, this will indicate that the change over time in outcomes differs significantly between the treatment groups. In sensitivity analysis to examine the impact of treatment intensity, we will also evaluate the effects of the intervention stratified by intervention dosage (frequency of interaction with the CHW, or mHealth or texting program).

### Secondary Analysis

Next, we will assess whether patient activation, self-management behaviors, biologic markers, and transfer to adult care are mediators of intervention treatment effects. This analysis focuses on the role of patient activation and self-management behaviors at 6 or 12 months as mediators of the effect of the intervention on the study outcomes. We will perform the mediation analysis using structural equation modeling; this will allow us to estimate the total effect of the treatment on outcomes as a sum of direct effects and indirect effects that are mediated by patient activation, self-management behaviors, biologic markers, or transfer to adult care. To evaluate the role of each potential mediating variable, we will calculate the proportion of the total effect that is mediated, the ratio of the indirect effect to the direct effect, and the ratio of the total effect to the direct effect. We also evaluate percentile and bias-corrected bootstrapped SEs and CIs for the effects.

We will next perform an exploratory analysis to identify individual and family factors that moderate intervention treatment effects. To test the hypothesis that adults with SCD and high disease severity will moderate the effect of intervention on outcome measures at 6, 12, and 18 months, we will first evaluate whether the impact of the intervention on overall changes (6 m minus baseline) differs between the high versus low severity groups. This will be tested by building a regression model with changes (end of intervention minus baseline) as the outcome variable, and that includes indicator variables for treatment groups, severity group (high vs low), and group-by-severity interaction terms. If the interaction term for a particular treatment group differs significantly from 0, this will indicate that the impact of that intervention differs according to (is moderated by) severity of disease. Next, to evaluate moderation in longitudinal models, we will fit models with change since baseline at 6, 12, and 18 months as the outcomes; these models will also include indicator variables for treatment groups, indicator variables for severity group, and time-by-severity interaction terms. If the interaction terms differ significantly from 0, this will indicate that the change since baseline depends on both treatment group and severity status (ie, that the impact of treatment is moderated by severity of disease). We anticipate that this model will be adequate to evaluate interactions; however, it may be necessary to consider more complex models, by modifying the longitudinal models for analysis of our primary outcome variable to include time by severity group, severity group by intervention, and severity by time by treatment group (3-way interaction) models. Finally, to explore the moderating effects of individual and family demographic characteristics on treatment effects, we will convert all patient and family demographic variables into dichotomous variables and evaluate potential moderation using the approach just described for severity status.

## Results

The COMETS study was funded in September 2017, received IRB approval in June 2018 (IRB #18-015106), and was registered in August 2018 (NCT03648710). The study is being conducted in accordance with the IRB-approved protocol (December 15, 2020). Participant recruitment began in January 2019 and ended in December 2022. A total of 405 participants were enrolled, with 374 being randomized to the intervention arms (enhanced usual care: 127, mHealth: 125, and CHW: 122). Data collection was completed in November 2024, with results expected to be submitted for publication in peer-reviewed journals in 2025.

## Discussion

### Principal Findings

This research aims to compare the effectiveness of 2 interventions, a CHW intervention and an mHealth intervention, to enhanced usual care alone. Our study seeks to address the significant gap in research focused on transition to adult care, while examining the impact of such interventions on chronic disease management, health care use, or health outcomes. The COMETS trial is one of the first multisite RCTs to assess the effectiveness of these interventions specifically for young adults with SCD.

Despite the under-studied effectiveness of mHealth and CHW programs in improving the transition to adult care for emerging adults with SCD, this research hypothesizes that incorporating either a CHW or an mHealth app will improve HRQOL and reduce acute care use. This trial aims to identify effective interventions to support young adults with SCD as they navigate the complexities of adult care, addressing gaps in current research. Positive results could translate into significant cost savings for payers and health care systems by reducing avoidable emergency department visits and hospitalizations, informing funding agencies such as Health Resources and Services Administration and State Title V programs dedicated to improving care for children with special health care needs. With the increase in interest and clinical recognition of both CHW programs [64,65] and mHealth apps [66-69], our study aims to support the growing body of research to aid in integrating these interventions into standard care models potentially leading to more personalized, accessible, and efficient care. This increase in mHealth app use and support for CHW programs in not only patients with SCD but also among patients with other chronic diseases is important to acknowledge. These interventions were chosen for their potential cost-effectiveness and scalability. Ultimately, demonstrating measurable improvements in health outcomes and cost savings will be key to integrating these personalized and accessible interventions into standard transition care models, leading to more efficient care and sustained improvements in health outcomes for young adults with SCD and other populations with chronic diseases.

### Strengths and Limitations

The study uses several key strengths to enhance its effectiveness and scalability. First, the involvement of stakeholders, particularly young adult coinvestigators, during the development phase ensures that research is aligned with their real-world experiences. This provides a comprehensive understanding that can inform implementation. In addition, building upon the established IMPACT program [40], the study leverages past research and implementation insights for further refinement and enhancement. In response to the limitations posed by COVID-19, such as reduced in-person recruitment opportunities, the study incorporates diverse communication methods to increase accessibility and engagement. Finally, the use of robust patient registries allows for real-time monitoring and identification of patients with SCD who meet study criteria, facilitating a streamlined recruitment process even with pandemic-related constraints.

SCD interventions have anticipated limitations that must be acknowledged, particularly when engaging young adults. Emerging adults are often at pivotal time in their lives where they might be transitioning into college or the workforce. This stage of life is inherently demanding and can make it challenging for young adults to adhere to treatment plans and prioritize participation in SCD programs. In addition, there is a widespread distrust of the health care system among this demographic, often rooted in historical and systemic inequities, which can be exacerbated by experiences of discrimination and stigma in both health care settings and broader society [70]. These factors together can lead to feelings of alienation and reluctance to engage with and adhere to SCD treatment plans or seek necessary care [70]. Additional limitations stemmed from the population being emerging adults, specifically surrounding the reliance on self-reporting and its potential bias. Recall bias is a challenge because adolescents and young adults with SCD may not accurately remember the past, or due to social desirability bias, they may answer questionnaires with what they think they should say or what they believe is socially desirable. To mitigate these challenges and bias, we carefully chose questionnaire designs and collected input and consulted adolescents and young adults with SCD and other stakeholders who could speak to these challenges. Furthermore, addressing these challenges requires targeted strategies that build trust through culturally competent care, increase accessibility by aligning program delivery with young adults’ schedules, and provide support systems that acknowledge and alleviate the unique pressures faced during this life transition.

In conclusion, the results of this study will contribute significantly to filling the gap in transition care research for emerging adults with SCD, guiding future intervention strategies, and providing valuable evidence for health care systems, payers, and policy makers. The potential impacts of these interventions extend beyond clinical outcomes, offering the possibility of improved psychosocial well-being, enhanced patient self-efficacy, and a more successful transition to adult care for individuals with SCD.

## Appendix

Multimedia Appendix 1 Sample size calculations.

Multimedia Appendix 2 Peer review report from PCORI.

## Abbreviations

CHOP: Children’s Hospital of Philadelphia
CHW: community health worker
COMETS: Community Health Workers and Mobile Health for Emerging Adults Transitioning Sickle Cell Disease Care
EMR: electronic medical record
GEE: generalized estimating equation
HRQOL: health-related quality of life
IRB: institutional review board
mHealth: mobile health
QLS: quasi-least squares
QOL: quality of life
RCT: randomized controlled trial
REDCap: Research Electronic Data Capture
SCD: sickle cell disease
SMART: social-ecological model for adolescents and young adults readiness to transition
