# Supplementary material for: The Community Health Workers and Mobile Health for Emerging Adults Transitioning Sickle Cell Disease Care (COMETS) Trial: Protocol for a Randomized Controlled Trial
Source: JMIR Res Protoc. 2025 Sep 4;14:e69239. doi: 10.2196/69239 (PMC12447008; doi:10.2196/69239)
Supplement: Multimedia Appendix 1 [file resprot_v14i1e69239_app1.docx]

***Sample size and power***. The original power and sample size calculations were modified to account for the pandemic. The PCORI funding agency approved the following plan on December 22, 2022. The final projected sample size will be 370 but will be divided into three time-periods: pre-Covid, during Covid, and post Introduction of Vaccine Covid. The projected sample sizes in Table 1 also take drop-out and loss to follow-up into consideration by assuming an approximate 10% drop-out rate. The retention rate was slightly better than anticipated, so that the final sample size is 375. We will adjust for COVID impact in sensitivity analyses by adding an indicator variable for pre-COVID vs COVID recruitment (with during/post-vaccination groups combined). An additional sensitivity analysis will be performed that will stratify by the three time-periods: pre-Covid, during Covid, and post-introduction of vaccine (as per Table 2).

***Table 1. Projected sample sizes by pandemic period and study site.***

| **Site** | **CHOP** | **Northwell** | **Cincinnati** | **St. Chris** | **CT** | **All sites combined** |
| --- | --- | --- | --- | --- | --- | --- |
| **Anticipated sample size pre-pandemic** | 38 | 27 | 13 | 1 | 0 | 79 |
| **Anticipated sample size during pandemic** | 61 | 31 | 18 | 37 | 19 | 166 |
| **Anticipated sample size post pandemic** | 55 | 8 | 13 | 15 | 34 | 114 |
| **Total sample size** | 154 | 66 | 44 | 53 | 53 | 370 |

Sample size and power calculations for the primary and stratified (sensitivity) analyses are provided in Table 2.

**Table 2. Power and sample size calculations for each aim. Calculations for the primary analyses are provided in column one. Calculations for the stratified analyses that will be conducted during each pandemic period (pre, during, and post) are presented in columns two, three, and four, respectively.**

| **Study Period and Sample Size** | **Entire Study (n=370)** | **Pre-Covid (n=79)** | **During Covid (n=166)** | **Post Covid (n=114)** |
| --- | --- | --- | --- | --- |
| **Power Calculations for Aim 1** | We have 80% power to detect an 8.3 point change in PedsQL SCD Module Total Score* and a 4.7 point change in PedsQL 4.0 CGS YA** Scale | We have 80% power to detect an 18.3 point change in PedsQL SCD Module Total Score* and a 10.4 point change in PedsQL 4.0 CGS YA** Scale | We have 80% power to detect a 12.4 point change in PedsQL SCD Module Total Score* and a 7.1-point change in PedsQL 4.0 CGS YA** Scale | We have 80% power to detect a 15 point change in PedsQL SCD Module Total Score* and an 8.6-point change in PedsQL 4.0 CGS YA** Scale |
| **Power Calculations for Aim 2** | The required sample size to achieve between small and medium alpha and small beta paths was 368 in Table 3 of Fritz & MacKinnon (2007)***, so that we have 80% power to detect between small and medium alpha paths and small beta paths with a sample size of 370. | The required sample size to achieve medium alpha and medium beta paths was 71 in Table 3 of Fritz & MacKinnon (2007)***, so that we have 80% power to detect between small and medium alpha paths and small beta paths with a sample size of 79. | The required sample size to achieve medium alpha and between small and medium beta paths was 116 in Table 3 of Fritz & MacKinnon (2007)***, so that we have 80% power to detect medium alpha paths and between small and medium beta paths with a sample size of 166. | The required sample size to achieve between medium alpha and medium beta paths was 71 in Table 3 of Fritz & MacKinnon (2007)***, so that we have 80% power to detect between medium alpha paths and medium beta paths with a sample size of 114. |
| **Power Calculations for Aim 3** | We have 80% power to detect a point 10.3 change in PedsQL SCD Module Total Score**** and a 5.9 point change in PedsQL 4.0 CGS YA Scale | We have 80% power to detect a 23 point change in PedsQL SCD Module Total Score**** and a 13 point change in PedsQL 4.0 CGS YA Scale | We have 80% power to detect a 15.5 point change in PedsQL SCD Module Total Score**** and an 8.8 point change in PedsQL 4.0 CGS YA Scale | We have 80% power to detect an 18.6 point change in PedsQL SCD Module Total Score**** and a 10.6 point change in PedsQL 4.0 CGS YA Scale |
| **Power Calculation (HTE - gender; recruitment site; disease severity)** | We have 80% power to detect an absolute difference in differences of 1.8 for males versus females for each intervention*****; 80% power to detect the absolute difference in differences of fo1.9 for patients with severe versus moderate disease for each intervention******; 80% power to detect an absolute difference in differences of 2.3 between site and intervention******* effects. | We have 80% power to detect an absolute difference in differences of 4 for males versus females for each intervention*****; 80% power to detect the absolute difference in differences of 4.1 for patients with severe versus moderate disease for each intervention******; insufficient sample size in some sites to perform simulations to detect size by intervention effects. | We have 80% power to detect an absolute difference in differences of 2.7 for males versus females for each intervention*****; 80% power to detect the absolute difference in differences of 2.7 for patients with severe versus moderate disease for each intervention******; 80% power to detect an absolute difference in differences of 3.3 between site and intervention******* effects. | We have 80% power to detect an absolute difference in differences of 3.2 for males versus females for each intervention*****; 80% power to detect the absolute difference in differences of 3.2 for patients with severe versus moderate disease for each intervention******; 80% power to detect an absolute difference in differences of 3.7 between site and intervention******* effects. |

* *The detectable change in PedsQL SCD Module Total Score was calculated using PASS 2021 sample size software for one-way analysis of variance [ANOVA] contrasts assuming equal variance to detect a non-zero contrast of means for detectable change C in PedsQL using an F test with a 0.0167 significance level, assumed power = 80%, and assuming changes of +C, 0, -C for the three treatment groups with a common standard deviation of 19.90.*

** *The detectable change in PedsQL 4.0 CGS YA Score Module Total Score was calculated using PASS 2021 for one-way ANOVA contrasts to detect a non-zero contrast of means for detectable change C in PedsQL using an F test with a 0.0167 significance level, assumed power = 80%, and assuming changes of +C, 0, -C for the three treatment groups with a common standard deviation of 11.32.*

**** The required sample sizes to achieve small (0.14), between small and medium (0.26), medium (0.39), or large (0.59) alpha and beta paths in a mediation analysis (assuming power = 80%) are provided in Table 3 of Fritz & MacKinnon (2007). We use the required samples for the bias-corrected bootstrap because this is the most powerful approach considered by these authors.*

***** The detectable change in PedsQL SCD Module Total Score was calculated using PASS 2021 for one-way ANOVA contrasts to detect a non-zero contrast of means for detectable change C in PedsQL using an F test with a 0.0167 significance level, assumed power = 80%, and assuming changes of +C, 0, -C for the three treatment groups with a common standard deviation of 19.90. The calculations differ from Aim 1 because here we assume that approximately 65% of the study population size will be considered to have severe disease based on CHOP registry estimates of those taking hydroxyurea or on chronic transfusion, yielding 65% of the sample size (per treatment arm) for Aim 1.*

****** Calculations were obtained using 1000 simulations in Stata statistical software of a regression model of outcome (PedsQL SCD Module Total Score, with standard deviation = 19.9) on indicator variables for each intervention (2 and 3), an indicator variable for male sex, and two intervention by male sex interaction terms (intervention 2 by male, intervention 3 by male). The data were simulated assuming changes of -12, 0, 12 for males in intervention groups 1, 2, and 3 respectively and changes of -C, 0, C for females in intervention groups 1, 2, and 3, respectively. The absolute value of the difference in differences (males vs females) is 12 – C for both intervention 3 versus 2 and for intervention 1 versus 2. There will be no heterogeneity of treatment effects by sex if the absolute difference in differences (12 – C) is 0. We assumed that the proportion male was 0.48.*

******* Calculations were obtained using 1000 simulations in Stata of a regression model of outcome (PedsQL SCD Module Total Score, with standard deviation = 19.9) on indicator variables for each intervention (2 and 3), an indicator variable for severe disease, and two intervention by severity interaction terms (intervention 2 by severity, intervention 3 by severity). The data were simulated assuming changes of -12, 0, 12 for males in intervention groups 1, 2, and 3 respectively and changes of -C, 0, C for females in intervention groups 1, 2, and 3, respectively. The absolute value of the difference in differences (severe vs non-severe) is 12 – C for both intervention 3 versus 2 and for intervention 1 versus 2. There will be no heterogeneity of treatment effects by severity if the absolute difference in differences (12 – C) is 0. We assumed that the proportion of severe disease is 0.45.*

******** Calculations were obtained using 1000 simulations of a regression model for outcome (PedsQL SCD Module Total Score, with standard deviation = 19.9). The data were simulated assuming changes of -12, 0, 12 for CHOP/Northwell site participants in intervention groups 1, 2, and 3 respectively and changes of -C, 0, C for non-CHOP/Northwell participants in intervention groups 1, 2, and 3, respectively. The absolute value of the difference in differences (severe versus non-severe) is 12 – C for both intervention 3 versus 2 and for intervention 1 versus 2. There will be no heterogeneity of treatment effects by site if the absolute difference in differences (12 – C) is 0.*
