# Supplementary material for: The Community Health Workers and Mobile Health for Emerging Adults Transitioning Sickle Cell Disease Care (COMETS) Trial: Protocol for a Randomized Controlled Trial
Source: JMIR Res Protoc. 2025 Sep 4;14:e69239. doi: 10.2196/69239 (PMC12447008; doi:10.2196/69239)
Supplement: Multimedia Appendix 2 [file resprot_v14i1e69239_app2.pdf]

PATIENT-CENTERED OUTCOMES RESEARCH INSTITUTE
SUMMARY STATEMENT
(Privileged Communication)

**Principal Investigator:**	David Rubin
**Organization:**	The Childrens' Hospital of Philadelphia
**Project Title:**	Community Health Workers and Mobile Health for Emerging Adults Transitioning Sickle Cell Disease Care (COMETS Trial)
**PCORI Funding Announcement:**	Management of Care Transitions for Emerging Adults with Sickle Cell Disease
**Review Cycle:**	2016: Cycle 3
**Request ID:**	R-1609-36631

AVERAGE OVERALL SCORE: 26

QUARTILE: 1

In-Person Review Discussion Notes:

Strengths:

- Reviewers were generally enthusiastic about the strong study design, dissemination plan, and patient-centeredness in this study.
- The interventions are guided by validated conceptual models, Chronic Care and SMART, which were authored by co-investigator Schwartz.
- The plan to stratify participants by age and disease severity is a major strength of the study design and will help identify which patients might benefit most from each intervention. This information is especially useful for payers.
- The sample size is justified and clinically meaningful. Assumptions for data analysis are based on previous research, and missing data issues are addressed satisfactorily.
- The recruitment plan is active and seems reasonable. Recruitment is comprehensively planned in the inner city and outer counties. The research team has demonstrated good retention rates in previous work with sickle cell patients.
- Patients with sickle cell disease chose the primary outcome (quality of life) and a comprehensive list of secondary outcomes, as well as endpoints of the study. Participant incentives are generous, which will likely enhance retention in the study.
- The highly qualified and complementary research team has a clearly defined leadership structure. Institutional support for the proposed research is excellent.
- The application describes diverse dissemination outlets, including church groups.
- The research team demonstrates meaningful engagement with diverse stakeholders—including members of Congress and policymakers—who were involved in developing the project.

Weaknesses:

- All weaknesses were deemed minor or fixable by the panel.
- The most pressing concern about the study design is the potential for contamination by randomizing at

the patient level. Patients randomized to different interventions could be seen within the same clinic. This was tempered by an understanding that health information is difficult to control, and that cluster randomizing is difficult with just three sites.
- The power analysis for Aim 1 is overpowered, and the power analysis for Aim 2 does not include the Root Mean Square Error of Approximation (RMSEA).
- There was some concern about the high proportion of available patients needed for the study, though this was tempered by the study team's history of retention.
- Information is lacking regarding the community health workers (CHWs) in the following areas:

- It is unclear how CHWs will be matched to patients and how often they will communicate with patients.
- It is unclear how the case load for the CHWs will be managed and how CHW costs will be covered, which raised concerns about feasibility and sustaining the intervention after the study concludes.
- It is unclear what happens to the CHW (and the app) after transition occurs.

- The intensive survey data collection, consisting of 11 survey instruments and over 200 items, could be burdensome to patients. There was concern about whether self-report measures are sufficiently objective.
- It is not clear if there will be enough adult providers to absorb the potential backlog of transitioning emerging adults.
- It is unclear how results from the study will be delivered directly to patients.
- It is unclear if the iManage tool has shown efficacy.

Human Subjects Protections:

When participants turn 18 years old, they will need to be re-consented.

Criterion 1: Potential for the study to fill critical gaps in evidence

<u>Reviewer 1</u>:

Strengths:

- The application convincingly describes the clinical burden of emerging adults with SCD (moderate).
- Compelling rationale. There is substantive evidence presented including a systematic review (Cochrane) of transition for chronic pediatric diseases that supports a gap in knowledge about how best to transition people with sickle cell disease from pediatric to adult care as well as for other chronic conditions (moderate).
- There are clear gaps in clinical knowledge as demonstrated by the variety of transition models implemented for people with sickle cell disease including those presented this in this proposal as well as nurse transition guides and in person training programs led by social workers, psychologists, and child life specialists (major).
- Clear gap identified by systematic review in SCD transition and all chronic illnesses of children in transition interventions (moderate).
- The investigators plan to compare two interventions that have been show to improve self-management and adherence to treatment and medical appointments in emerging adults as well as process measures associated with improved care and decreased utilization in some studies. There is convincing evidence that both patients and caregivers identify lack of information about transition as one of their most important concerns, and that this research could potentially answer these gaps in knowledge (major).

Weaknesses:

- Overstatement of burden of mortality in emerging adults vs. school age and older adults with SCD. This reviewer would challenge the statement that there is a 7-fold increase in mortality for people 16 to 25

years of age with SCD. At the very least, the comparator group is unclear. If it is AA without sickle cell disease, increases are also seen in the 5-14 and 25-34 age group of similar magnitude (minor).
- Relatively limited data supporting the use of these or other interventions to improve the quality of transition (moderate)
- Relationship between effective transition and HRQL is not convincing in background (minor).

Reviewer 2:

Strengths:

- The application gives evidence about the clinical burden and the gaps in current knowledge through the acknowledgement of poor outcomes and systematic reviews that fail to include studies on young adults and patients with SCD. The information is concise but convincing. This is a moderate strength and will contribute to focusing the outcome.
- The application supports the potential to fill the evidence gaps by detailing and focusing on the concepts in the Chronic Care Model and the SMART Model in terms of self-management. It emphasizes that the importance of self-management is critical to staying healthy and chooses programs focused on self-management. This is a major strength that will lead to improvements in healthcare because it identifies an important concept to filling the evidence gaps and chooses to focus the research on this concept.

Weaknesses:

- Though the application provides a real-world patient example of the lack of clarity on what method of transition should be explored, it does not identify the gap through the lens of clinicians and their decision making. This is a minor weakness because it will not substantially impact the outcome and inconsistencies at the clinical level can be assumed.

Reviewer 3:

Strengths:

- Proposal provides detailed description of the overall clinical burden of sickle cell disease among children, adults, and those transitioning in between the age groups. Data is provided for mortality, morbidity and quality of life measures
- Critical gap identified in the proposal points to lack of evidence and research supporting interventions that reduce morbidity and mortality during transition period. Specifically there is no knowledge about the efficacy of community health workers and mobile health applications on self-management of sickle cell disease.
- If carried out successfully the study would have the ability to describe what kind of effect does the utilization of community health workers or mobile health applications have on the quality of life measures as well as acute care utilization, relative to enhanced usual care.

Weaknesses:

- Proposal should provide more detail on the clinical burden in the local target area and among the minorities (minor)

Reviewer 4:

Strengths:

- Major: The application begins with an anecdote that effectively and clearly establishes the problem and the central study question. In sickle cell disease (SCD) patients transitioning from pediatric to adult care settings, which interventions will improve patient self-management, health outcomes and best facilitate the care transition.
- Major: The study team comprised of researchers, clinicians and social scientists from Philadelphia, PA and Cincinnati, OH has demonstrated thorough understanding of the field of sickle cell disease and its clinical and structural care gaps, particularly as these gaps relate to care transitions for people aging out of the pediatric health system. The team has outlined a strong and comprehensive justification for collection of evidence related to care transitions for sickle cell disease patients.
- Moderate: The literature lacks sufficient evidence to support best practices to facilitate transition of sickle cell patients from pediatric to adult care and the study design has been crafted to address this challenge. The team proposes to assess the effectiveness of two interventions- community health workers (CHW) vs. mobile technology, in facilitating care transitions. The CHW intervention has demonstrated efficacy in improving outcomes in chronic diseases like asthma, diabetes, hypertension and HIV. Mobile health platforms have been shown to improve self-management and adherence in diabetes and liver transplantation. Given these successes, the study team proposes to assess the efficacy of these interventions in improving health outcomes for SCD patients who transition from pediatrics to adult care settings.

Weaknesses:

- No notable weaknesses. Team has thoroughly addressed gaps in evidence with over 150 references cited.

Reviewer 5:

Strengths:

- Strong evidence of a significant clinical burden is presented including the great increase in mortality during transition years, dramatic increase in ED visits and hospitalizations. Major
- Evidence of a critical gap was demonstrated using the Cochrane review. Limited evidence that exists in other diseases was presented and provided sufficient justification for comparing comparing community health workers and a mobile app with standard of care. Major
- A large evidence gap in SCD transition interventions was demonstrated. Given the lack of high level evidence in SCD, it is not possible to recommend either proposed interventions. Major
- Strong support is given as to how findings from this study would inform a wide range of stakeholders including patients, families, healthcare providers, payers and policy makers as to which intervention is more effective. Major

Weaknesses: None noted.

Criterion 2: Potential for the study findings to be adopted into clinical practice and improve delivery of care

Reviewer 1:

Strengths:

- The investigators effectively communicate a plan to adopt these interventions into practice by effectively working with local, regional, and national stakeholders. They have identified community-based organizations, affected individuals and their families, third party payers, government, and health care providers as the relevant stakeholders and their role in advocating, funding and implementing the study findings. They do not specifically mention guideline development or interaction with the American Society of Hematology or the American Society of Pediatric Hematology/Oncology, or the Sickle Cell Adult Provider Network, but do have a stakeholder from the American Academy of Pediatrics (moderate).

- They have several surveys that support the need for transition information for patients and their caregivers ad local survey data on the importance of this topic with providers of pediatric sickle cell care (major).
- Scalable study interventions (moderate).
- Dissemination plan that includes third party payers and advocacy (major).
- Funding for community health workers from Keystone Health (moderate).
- A study that demonstrated improvement in the quality of care with these interventions would drive uptake of these interventions if there is effective advocacy to fund these relatively expensive interventions and engage patients that are not receiving incentives to participate (moderate).
- The major barrier to widespread implementation include the cost and local expertise needed to develop community health worker or implement and support the mobile health application. There is a moderately detailed implementation plan that includes the engagement of policy makers and funders (moderate).

Weaknesses:

- Cost of interventions (minor).
- No plan to transition to effective engagement of emerging adults without study incentives (minor).

Reviewer 2:

Strengths:

- Study findings will be disseminated through the local and national stakeholder organizations led by their committee to ensure that results reach patients, parents, church groups, and policy makers. The application provides an extensive, diverse list of outlets that will disseminate findings. This is a major strength.
- The application does identify that stakeholder groups who wanted more information about the use of community health workers or mobile health initiatives will be the end users. Self-management is a main concern of patients and parents and the focus of the findings will emphasize which method will best foster self-management. Providers will be able to identify which technique would best serve their patients in transition. This is a moderate strength because it shows that results are tailored to those who need it the most.
- Along with the release of the findings through an Evidence-to-Action brief, the researchers will make the mobile health application available and provide a community health worker manual to reproduce the findings. This is a moderate strength; having the tools available will probably improve healthcare.

Weaknesses:

- The application does not provide a detailed strategy to engage all of the end users that are provided in the list, only that the information will be made available for groups to share. This is a minor weakness because it does discuss that it will be worked on with the stakeholders' input.

Reviewer 3:

Strengths:

- Primary health decision makers are identified as patient stakeholders that are transitioning from adolescent to adult care. However the health decision making in this case is also relative to families, providers, agencies, payers, and policymakers.
- There is a documented history of patient and stakeholder involvement in the study planning, both patients and providers identified this study as a needed step in closing a research gap for those transitioning care.
- Positive research findings from this study would inform patients about an intervention approaches that would perhaps improve their quality of life and disease self-management as well as reduce morbidity outcomes compared to the standard of care.

- It appears likely that results and implementation could be easily reproduced by others. Community health workers are commonly embedded in sickle cell management teams or local agencies and mobile health applications are available from app stores or internet. Implementation would depend on the pool of available and trained community health workers, which is a potential barrier.
- There is a comprehensive dissemination plan in place that spans from local and stakeholder levels to national level.

Weaknesses:

- Efficacy of interventions involving community health workers and mobile health in disease self-management has already been demonstrated in numerous other disease areas. Why would it be any different with sickle cell disease? (minor)

Reviewer 4:

Strengths:

- Moderate: A variety of stakeholders is identified, including SCD patients. Tailored explanations are provided about how study results will benefit each stakeholder group. For example, the challenge many transitioning SCD patients experience is lack of information about HOW to transition care. The proposed interventions can serve as sources of support and information delivery to facilitate timely and efficient linkage to a new provider and consistent engagement in care with this provider.
- Moderate: The study justification arises from clinical expertise and patient voices who define the ongoing problem.
- Moderate: Demonstration of the effectiveness of care coordination and/or mobile technology can lead to shifts in program approaches for this population. The study may also assist in identifying which patient characteristics are amenable to care coordination vs. mobile technology support.
- The dissemination plan is comprehensive with respect to stakeholders.
- Because the interventions are accessible, community health workers (CHW) and mobile app, there is a high likelihood this intervention could be easily adopted in care settings across the country.

Weaknesses:

- Moderate: The dissemination plan lacks detailed description of how the information will be provided directly to patients in care rather than through advisory bodies and traditional communication portals.
- Moderate: The ability to modify or enhance program staff needed to implement these interventions require funding for personnel and training. Publicly-funded insurance programs like Medicaid and Medicare have created allowances for reimbursement for teleheath which, depending on the state, could be a revenue source for the mobile health intervention but probably not app-based interventions. CHWs are not yet a recognized service provider billable to Medicaid or Medicare. Demonstrating effectiveness of these interventions could produce critical information needed to shift national policy toward reimbursement for these services.
- Major: Since program implementation or program behavior change is often driven by reimbursement or compensation, absent grant funding, the incentives and supports for adopting these interventions once proven effective may be absent. This ultimately affects the ability and will to scale and replicate these interventions. However, study findings may provide evidence needed to direct and inform health insurance policy decisions, particularly as the field is moving toward value-based payment models.

Reviewer 5:

Strengths:

- Stakeholders, or decision makers, include adolescents, family members, providers, payers, federal and state funders, and policy makers. Congressmen, policy makers, patient advocacy organizations all play

a role in these decisions and were appropriately identified. This project will provide high level evidence to determine the effectiveness of both interventions compared with usual care. All of these stakeholders are also end users and strategies to engage were elaborately described and included in LOS as well as dissemination plans. Major.
- The application provides strong support for the RCT and choice of interventions from the SCD community. SCDAA just launched a CH worker online training program of 80 hours that will result in a certificate. This supports the need for validated CH workers as an intervention. Major.
- The data will include both patient outcomes such as QOL, important to patients and families, health services use important to payers and legislators, and clinical data important to providers and patients. Based on this data, if a positive trial, providers would be able to order CH or mHealth interventions that ultimately payers would reimburse. The impact for decision makers will be great. Major.
- A very strong dissemination plan is described beyond usual publications and conferences. Several patient advocacy groups are involved in the advisory board and will promote the results. A mobile app and CH worker manual, as well as transition readiness tools, will be made available for dissemination upon project completion. Moderate

Weaknesses: None noted.

Criterion 3: Scientific merit (research design, analysis, and outcomes)

<u>Reviewer 1</u>:

Strengths:

- Underlying model for intervention development and experience with the interventions. The application proposes using two conceptual frameworks: the Chronic Care Model and the Social-Ecological Model of Adolescents and Young Adults Readiness to Transition (SMART model developed by a co-investigator with limited preliminary data in pediatric cancer patients (major).
- Methodology: Rationale for individual participant randomized controlled trial (non-clustered) and impressive training plan and evaluation of fidelity of CHW intervention. The outcome measures are well validated and would add absolute neutrophil count to markers of disease control as target to guide hydroxyurea dosing. (moderate)
- Medicaid data on 30% subset of patients from CHOP with Keystone MCO (moderate).

Weaknesses:

- Regarding feasibility of randomizing 450 emerging adults over 3.5 years of active enrollment from only 3 centers: major concerns about the effective recruitment from the pool of 450 patients >17 years followed at the 3 centers as they propose to intervene and transition all of these patients within 12 months of starting the intervention. Some of the patients may not be ready to transition for several years developmentally. The year age cohort is about 75 people, so this implies a back-log of 5 years. Will the adult providers be able to see the increased numbers of patients prepared for transition? Also 450/630 enrolled is 71% participation, a very high proportion for a RCT in SCD or most other disease states (major).
- The enhanced usual care group should include measurement of transition readiness and a transition summary, not just provision of medical records. This is based NHLBI guideline recommendations (moderate).
- Generalizability is limited. Study setting is limited to mostly urban and suburban patients (minor).
- Methodology: this reviewer is confused by the measurement of monthly exchange transfusion, but no mention of simple transfusions as a treatment option. Also how will patients randomized to Mhealth without smart phone or tablet access be analyzed? The schedule of enrollment leaves time for only follow-up for 9 months for the last enrolled patients. Also, there is concern about stratification for age and severity in 3 sites (including two smaller sites, may lead to some empty cells). Hydroxyurea is not a very good indicator of severity given increased use in many programs (minor).
- This reviewer is curious about how the investigators plan to approach HRQL in patients that have stayed in pediatric care (may be better) vs. left pediatric care at 12 months. How likely is it that these interventions will change HRQL by 10 points-would it provide a clear description of effect sizes in other studies? (moderate).

- Some assumptions such as data missing at random are unlikely to be true. The investigators are likely to be able to detect interaction between treatment and severity especially if there are few people in the less severe group. There is potential confounding by severity and frequency of engagement with intervention and routine clinical follow-up that is not addressed (minor).

- The study evaluates transition to adult care as a mediator as the effect, but HRQL may improve with intervention and no transition to adult care (minor).

Reviewer 2:

Strengths:

- The aims of the proposed research are supported by discussions with stakeholders, especially patients who agree that quality of life and self-management are important outcomes. The conceptual frameworks are clear and rooted in self-management. This is a major strength.
- The patient population and study setting are appropriate; the researchers keep in mind that a more urban population like Philadelphia will be able to recruit more patients than the other recruitment sites. The sites are diverse in terms of population and setting. This is a moderate strength.
- The application discusses issues with patient attrition in detail and builds upon a past study that was successful as well as the fact that many of the patients recruited have been in prior research studies. This is a moderate strength and could lead to improvements in outcomes because it seems that patients will most likely continue participation in the research study.

Weaknesses:

- There does not seem to be a formal way for participants to provide input on the study, especially the specific community health workers and the technology in the study. This is a minor weakness because it could be easily added.
- The application and study design does not address a way to wean patients off of the mHealth technology and community health worker to move towards self-management. This is a moderate weakness because it would limit the impact of healthcare in a practical setting.

Reviewer 3:

Strengths:

- Study is conceptually based in both the chronic care model and social-ecological model of adolescents and young adult's readiness to transition. This model identifies the variables related to patient, families and providers that contribute to transition readiness. Aims are based on this model. Both models have been validated.
- Study design is a three arm individually randomized approach. Randomization is block stratified and evenly distributed between the study arms. There are four repeated outcome measurement time points.
- Primary end point for the study is identified as patients quality of life. It is appropriate and justified, and it was selected by patient stakeholders as the most important outcome measure. Other outcomes include disease self-management and acute care utilization.
- Patient population and setting are appropriate for the questions and aims of interest.
- Comparator arms are well described and justified. Enhanced standard of care comprises mainly of health education strategies which are common to all participating locations. New intervention arms add either a community health worker or mobile health application to the enhanced standard of care.
- Sample size is estimated based on the proposed design, and the effect size is justified and clinically meaningful, at 10 point improvement in the quality of life scale. Assumptions such as standard deviations of the difference have been justified based on previous research.
- Analytical plan is detailed, appropriate and well described.
- Missing data problems are addressed through available analytical methods.
- Recruitment which is divided between the participating centers is reasonable.

Weaknesses:

- There is a discrepancy between 450 enrolled subjects and plan to evaluate or analyze 360. Further justification is needed about the difference of 90 subjects. (minor)
- Enrolling 450 subjects out of a pool of 630 seems optimistic and raises feasibility concerns. (minor)
- Aim 1 outcomes seem a bit overpowered at 0.97 and 0.99, respectively. Sample could be reduced or proposed detectable effects could be smaller at some acceptable power level. (minor)
- Power for Aim 2, structural equation model, could be evaluated via root mean square error of approximation (RMSEA), a fit measure between the theoretical and fitted model. (minor)
- It would be interesting to see a direct comparison of community health worker and mobile health interventions (minor)

<u>Reviewer 4</u>:

Strengths:

- Moderate: The theoretical framework is clearly delineated and a team member is a co-author of and experienced with the framework.
- Major: An RCT design assessing the impact two timely interventions is the backbone of this study and can provide the most rigorous evidence possible.
- Moderate: The study plan is feasible and was conceptualized by seasoned investigators who have conducted similar studies. The engagement milestones 6, 12 and 18 months are appropriate but will only provide insights about short-term impact. (See weaknesses).
- Moderate: The SMART model has demonstrated effectiveness in other conditions and should be amenable to testing in a different disease state
- Major: Assessment of the impact of technology on patient outcomes is timely and results of this may have far-reaching impact not just in SCD but may prove to have applicability across the health sector and in other diseases.
- Major: The recruitment strategy is feasible and comprehensive and includes several steps from identifying patients before, during and after clinic visits to conducting face-to-face visits during hospitalizations. The team has significant experience from 14 previous research trials with ensuring retention and noted study retention rates of up to 80%.
- Minor: Subgroup population analysis potentially can provide important insight.

Weaknesses:

- Moderate: Given three-arm randomization, the sample size may be too small to detect true and practical differences in sub-group analyses.
- Moderate: The final assessment is at 18 months which will not provide insight about the long-term impact of the intervention.
- Moderate: Self-report measures are often fraught with bias and unreliability. They also are subject to social desirability bias which results in study subjects providing untruthful or exaggerated responses because they either want to please the investigator or provide an answer that is viewed favorably by others. These responses can bias study findings making it difficult to discern the true impact of the intervention. In some cases, as with this team's quality of life data collection component, subjective answers can not be avoided and this is difficult to account for in study interpretation.

<u>Reviewer 5</u>:

Strengths:

- The Chronic Care Model and Social Ecological Model for Adolescents Readiness to Transition (SMART) was used to guide the project and was supported by the literature. The intervention supports self-management which will have an effect on study outcomes including health services use, QOL, patient self-efficacy, and clinical indicators of health status. Major
- A pragmatic RCT was chosen vs. cluster RCT. A good rationale was provided in that the geography of

the centers would not prohibit the use of a RCT. The interventions will be delivered outside of the healthcare team. This is a strong design. Moderate

- Multi-center, pragmatic RCT comparing mobile app or CHW vs. usual care on 6, 12, and 18 month patient-centered and health services utilization. Usual care will include a social worker consult which provides some baseline assessment/intervention for all. The comparators, mHealth vs. community health worker vs. enhanced usual care (+social worker) is very justified. These interventions have been used in other diseases for transition with some evidence, and insufficiently tested in SCD transition. Major
- Sub-analysis will determine whether each intervention is more effective in a particular sub-group such as disease severity, age, or socioeconomic status. Adolescents with more severe disease and those who are older are more likely to have more negative outcomes and a higher disease burden to manage. Subjects will be stratified upon enrollment based on age and disease severity. The criteria are justified. This analysis will help stratify selection criteria for the intervention if differences are found. This could be important to payers. Major.
- All centers have successfully recruited between 78-83% of patients in previous trials. Sites have a strong track record of successful recruitment. A variety of recruitment and retention methods are used, strengthening the likelihood of good recruitment and retention. Major
- Dr. Smith-Whitley at CHOP has a real-time registry that identifies when patients will be in clinic. Dr. Szalsda has an electronic system that alerts providers to upcoming visits. These in-place systems will greatly increase successful recruitment. Major
- Incentives for participation were determined by patient members of the study team and will encourage retention. Major
- Stratified randomization based on site, disease severity and age (17-21 vs. 22+) is well justified due to differences in outcomes. Moderate
- Will enroll 450 for usable data for 360. Given history of recruitment and planned recruitment and retention plans, this is sufficient. Moderate
- Power analysis were presented for Aims 1 and 2 and justified, and demonstrate sufficient power to answer primary outcomes. Aim 3 is appropriately described as exploratory. Moderate
- PCORI methodology standards are adhered to. Major

Weaknesses:

- A rationale for the defense of a 6 month intervention is not provided. Moderate
- The number of CHWs that will be hired is not clear. A vague description of their role or number of expected interventions per patient is not provided. There does not seem to be sufficient support in the budget (10% at CHOP, $0 at other sites) to support the intervention. Thus feasibility of this intervention is not entirely clear. Moderate.
- A very brief description of the mHealth app was provided. Moderate
- Definitions of severe disease were different within the application. One definition included HU and monthly transfusions, and another did not.
- While interventions are similar, the iHealth intervention also includes daily tracking of symptoms. Minor
- The number of patients surveyed about selecting important outcomes was not defined, “a number of…”, “…the majority responded….) Minor.

Criterion 4: Investigator(s) and environment

<u>Reviewer 1</u>:

Strengths:

- Experienced core team with established relationships. The team of investigators particularly at CHOP and Cincinnati Children’s Hospital are very well qualified to complete the study with clear evidence of clinical and statistical expertise. The study structure with a more junior investigator (currently an Instructor, responsible for the practical implementation of a study this size with mentoring is well described with clear roles (moderate).

- Complementary disease specific knowledge/research and policy expertise of dual PIs. Rubin’s experience includes study methodology, health services research, and advocacy, and Dr. Smith-

Whitley's includes clinical sickle cell disease, leadership of clinical program, and co-investigator on clinical trials and some of the needs assessment studies in emerging adults with SCD. One of the two dual PIs has experience in leading studies of nearly this size and complexity and the level of effort is appropriate for the investigators and study personnel (major).

- Excellent institutional infrastructure for research including access to a large patient population and resources to aid in dissemination of results and inform policy development. There are excellent letters of support from the departmental leadership and the appropriate facilities and resources except for a relatively limited number of potential participants. All letters of institutional support are very positive (major).

Weaknesses:

- Junior investigator with limited experience in sickle cell disease. This third PI responsible for implementation which may lead to differences in leadership and challenges in recruitment as implementing PI has no current role in SCD. Dual PI with sickle cell expertise has predominantly been a co-investigator, not project leader for research studies (minor).
- The adult hematologists have clinical expertise, but little experience in clinical research and limited involvement of adult providers--one adult sickle cell provider at U Penn and a consultant--while many patients transition to other hospitals (minor).

Reviewer 2:

Strengths:

- The dual-PI structure presents complementary insights on the project, Dr. Rubin has the population health and large-scale study experience while Dr. Smith-Whitley is able to provide expertise on sickle-cell disease in this population. They have a history of collaboration and understanding of the study population as evidenced in their resumes. This is a moderate strength and could influence the accuracy of the study procedures and thus the outcomes.
- The study has access to experts on mHealth and community health workers through collaborators like Dr. Szalda. This organized structure ensures that both study arms are given equal support. Thus, this is a major strength.
- Renowned institutions like the Comprehensive Sickle Cell Center will be supporting the research. This is a major strength because the research will be exposed to experienced personnel from these centers to conduct research that will influence healthcare.

Weaknesses: none noted.

Reviewer 3:

Strengths:

- This is a Co-PI lead project and PIs have complementary expertise, in pediatric hematology and oncology, and population health management. They have a history of collaboration. Their roles are separate with scientific and project oversight. Both PIs have a record of leading projects of similar size, duration and scope.
- Research team is diverse, and inclusive of various experts and backgrounds, for example, internal medicine, hematology and oncology, pediatrics, psychology, clinical epidemiology, biostatistics, and patient and community partners.
- Research team members are qualified and experienced in this area of research.
- Clinical and research resources at all participating sites are suitable to carry out the project and provide access to the target population.
- Institutional support and additional resource commitment based on the letters will increase the probability of success and provide suitable environment.

Weaknesses:

- none noted.

Reviewer 4:

Strengths:

- Major: Numerous investigators with research and clinical experience, including team members instrumental in design of tools and methodologies, are incorporated in the RCT. Study investigators and stakeholders together create a beautifully diverse and research-experienced team.
- Moderate: Multi-disciplinary nature of the team can enhance implementation and use of findings post study period.

Weaknesses: None noted.

Reviewer 5:

Strengths:

- Co-PIs bring expertise in policy, CBPR and SCD. A strong multi-disciplinary team has been assembled for this project including CBOs and policy stakeholders, patients, parents, social workers and providers as Co-Investigators. Major
- Dr. Rubin has expertise in HSR, population health and policy. Dr. Rubin will be responsible for engagement of payer stakeholders, and strategies for dissemination accounting for policy. Major
- Dr. Smith-Whitley is a nationally recognized expert on SCD and former CMO for SCDAA. Rubin and Smith-Whitely have complementary experience as DPI's for the project. Major
- Together the team has demonstrated the ability to conduct similar projects of size and scope. Major
- A senior Professor is included as a Co-I to guide and conduct the statistical analysis. Major
- CMOs of the state Medicaid for both OH and PA are on the Stakeholder Advisory Group. Other members of the SAC are very strong. This is important because it will help influence dissemination and payment for the interventions if found to be efficacious. Major
- There is an extensive list of letters of support indicating strong support from each center, patient stakeholders, congressmen, policy and payors, as well as institutional, PI and Co-PI support. CHOP is dedicating additional resources to start a SCD transition registry. Major
- There are 4 major centers involved in this project: CHOP, U Penn, St. Christophers and University of Cincinnati Children's Hospital. These are the only centers in the geographic areas of Philadelphia and Cincinnati that refer adolescents with SCD for transfer to adult programs. Combined, the centers have a sufficient population to recruit successfully for this study. All centers have very strong SCD programs. Major
- Investigators seem to have sufficient effort. Major

Weaknesses:

- It appears that there are no patient representatives from Cincinnati. Suggest adding one. Minor.
- It is not clear if there are sufficient funds to support the CH worker intervention and RA support at each site. Moderate

Criterion 5: Patient-centeredness

Reviewer 1:

Strengths:

- Patient surveys to identify priority outcomes. The applicants provide strong evidence that transition is of great importance to patients, caregivers, and other stakeholders and that evidence to guide

interventions to improve the quality of transition are a high priority (major).
- Demonstrated importance of preparation for successful transition to patients and caregivers. Patient and caregivers care about effective transition and consider quality of life and self-management of their disease as important goals and describe transition success as managing their own disease. This study will measure HRQL as the primary outcome and will also look at mediators of HRQL including transition to adult care (major).
- The CHW and Mhealth/texting intervention for transition has been implemented in other diseases and increasingly available, but are not usually covered by third party payers (minor).

Weaknesses:

- No discussion of alternative potential interventions to improve transition and HRQL in this population (moderate).
- Failure to discuss patient barriers to CHW interventions (minor).

Reviewer 2:

Strengths:

- The application emphasizes that interventions are becoming available in both healthcare and outside of healthcare settings. It also discussed that material will be provided to make sure others can use the mHealth and community health worker interventions. This is a moderate strength.
- The application explains that patients and providers do want answers to improve self-management after transition. It outlines the most important parts of the transition to patients, for example how to manage a chronic illness and live a normal life, and verifies these identified components with their stakeholder participants through surveys. It describes how these components will be addressed to close the evidence gap and make specific healthcare decision about the best method of transition. This is a major strength.

Weaknesses: none noted.

Reviewer 3:

Strengths:

- Application identifies that during the planning process, all stakeholders but primarily patients identified quality of life and disease self-management as the most important primary measures. In addition, acute care utilization was identified by provider and payer stakeholders as important.
- Application also provides the evidence that closing the knowledge gap and having better intervention options while transitioning to adulthood with sickle cell disease is important to patients, caregivers and providers and would give them critical information in order to make the best health care decisions.
- Standard of care which is common to all participating centers as well as other centers mainly consists of visit with the physician and social worker, information on health insurance, and identification of future providers. Other treatment transition options proposed here lack evidence and research regarding the sickle cell hence this is study is attempting to contribute to that area by comparing their effectiveness to enhanced standard of care.

Weaknesses:

- None noted.

Reviewer 4:

Strengths:

- Major: The study delineates specific clinical and social outcomes of interest relevant to improving care and transition from pediatric to adult care for the SCD population. Both the literature and a wealth of clinical experience demonstrate an urgency for identifying solutions for eliminating care gaps for SCD patients transitioning to adult care. Oftentimes, these care gaps are due to lack of social support and lack of information and understanding needed to link to a new provider and remain in care. The proposed interventions will both provide resources to help facilitate this transition.
- From a payer's perspective, these and any effective interventions are welcome because of their potential to reduce the frequency of emergency room visits and avoidable hospitals admissions. Evidence is needed to support payer decisions.
- Moderate: Inclusion of health literacy assessment demonstrates understanding of importance often overlooked predictor of health outcomes.
- Major: The recruitment plan was developed in conjunction with patients, well-conceptualized with aggressive approaches to active vs. passive recruitment. For example, recruitment efforts for many studies often consist primarily of marketing with flyers and word of mouth. This study team has outlined a multi-pronged recruitment approach which includes survey appointment rosters for eligible patients, reaching out to them before the visit and during the visit. They have also included a step involving making a personal visit to hospitalized patients to inform them about the study.
- Minor: The availability of a mobile app to facilitate care transitions and support is useful for sites who wish to replicate this intervention. Since mobile technology has been previously demonstrated to improve health outcomes in diabetes and heart disease, this intervention could easily be replicated now. However, the availability of and support for CHWs is variable but if available could also be used now to support care SCD transitions.

Weaknesses:

- Moderate: The number of instruments and queries may be burdensome for study participants. For example, there are at least 11 different instruments and scales/measures with 237 items patients need to complete at times: baseline, 6 months, 12 months and 18 months. The study team believes administration of these tools will require only 45 minutes to complete but this seems unrealistic, particularly for those with low reading literacy. If each of these variables is not essential, the team should consider prioritizing those which are definitely needed to demonstrate efficacy and answer the study question.
- Major: The list of dissemination stakeholders is expansive but there is no concrete plan outlined to communicate directly to patients with SCD other than through organizations, key opinion leaders and social media. Such a multi-pronged strategy as outlined is necessary but given the unique and longstanding nature of this problem--losing SCD patients when they age out of pediatrics-- face-to face, hands on communication about the study results, implications and use should directly reach patients, particularly study participants and other patients with SCD. Advisory councils are a great addition to study teams and play a valuable role but often the results are disseminated in impersonal mechanisms like websites, listserves and newsletters which patients don't read, thereby never receiving direct information about how the results can be useful to them.

Reviewer 5:

Strengths:

- There is an excellent discussion of why outcomes were chosen and that they were supported by patients. The team validated qualitative work with a sample of their patients who were asked to indicate which outcomes are most important to them. Patients indicated managing their disease and a good QOL as most important. The intervention supports self-management. Outcomes include SCD self-efficacy, knowledge and measures of overall QOL. It is clear that the outcomes selected by patients were included as study outcomes. Major.
- The interventions are available to some patients in some centers, but not universally. SCD patients have endorsed the desire to use both mHealth and community health workers in SCD. Major.

Weaknesses:

- There is no discussion of why closing this gap is important to patients and other stakeholders. Minor.

Criterion 6: Patient and stakeholder engagement

Reviewer 1:

Strengths:

- Well defined patient/provider involvement in the project as well as a multidisciplinary team. This application has clear documentation of the involvement of most of the relevant stakeholders in this project especially patients, providers, scientists, and third party payers. There is a well-documented plan for continued input from all of the relevant stakeholders and a well-developed engagement plan (major).
- Engagement through direct involvement of multiple patient advocacy groups (moderate).
- The inclusion of patients as co-investigators with support for their time as well as a multidisciplinary team and advisory board with meetings every 6 months should provide continuous engagement of other stakeholders (major).

Weaknesses:

- More limited involvement of training institutions, professional societies (minor).
- The roles of the study partners are all well defined, but the contribution to decisions is unclear with the exception of clearly defined processes for reaching consensus between the dual PIs and co-PIs. Limited discussion of how to reach consensus if there are divergent opinions in the larger advisory board (minor).
- It is unclear if the public sector stakeholders have had substantial input on the formulation of questions or identification of outcomes (minor).

Reviewer 2:

Strengths:

- Active engagement with stakeholder groups is detailed; the groups planned the study and the trial outcomes were selected by emerging adults. The engagement is reinforced through biannual stakeholder groups meetings that will ensure that input is given throughout all steps of the study. This is a moderate strength.
- The study focuses on emerging adults and will be able to engage them through technology and peer mentorship. These resources are appropriate to emerging adults in this day and age. Stakeholders will stay engaged through the dynamic nature of the study. This is a moderate strength because relevancy could improve attrition and then outcomes.

Weaknesses:

- The stakeholder group seems professional and scientist-heavy with only a few patients represented. The patient perspective could be more prominent with more patients as stakeholders. This is a minor weakness and could be easily addressed by approaching more patients to become stakeholders.
- The number of researchers involved as co-investigators, especially those without experience with sickle cell disease, seems cumbersome instead of necessary to patients. This is a moderate weakness and could become an issue with patient trust.

Reviewer 3:

Strengths:

- Research and stakeholder team is quite diverse and includes various stakeholders, patient and family members, providers, payers, policymakers, churches and community groups. They have been involved in all aspects of the study development and planning, and there is a plan of their continued involvement is study execution and dissemination.
- Engagement plan is detailed and it describes all stakeholder groups as well as their roles. There is a number of stakeholder on the research team in a role of Co-I, and the others are on the advisory group with appropriate compensation.
- There is a plan for all stakeholders active engagement in the study and for the dissemination of the results on various local and national levels and groups.

Weaknesses:

- None noted.

<u>Reviewer 4</u>:

Strengths:

- Major: This team includes seasoned researchers with extensive experience conducting clinical trials. For example, PI Dr. Smith-Whitely has participated in 14 clinical trials of similar complexity, and the other investigators have participated in at least nine trials. All three sites have previously recruited nearly 80% of their SCD patients to participate in research studies.
- Major: A co-investigator is an SCD patient and input has been obtained from patients throughout the process to inform this study.
- Major: Efforts were made to engage wide range of stakeholders across sectors and including patients and community.
- Moderate: Inclusion of a parent is also a valuable innovation and will likely yield practical and actionable insight.
- Moderate: These study sites appear to have appropriate organizational structure and foundational resources needed to conduct the study and engage patients. This capacity has been demonstrated by numerous previously-conducted studies.

Weaknesses

- Moderate: There are three PIs listed and the narrative denotes numerous “co-investigators” but the detailed roles and responsibilities of each person mentioned as a co-investigator could be better defined. Perhaps the notation of a co-investigator is thought to bolster the application by giving a sense of breadth and inclusion on the study team, but if each person cited is a co-investigator rather than a stakeholder or key informant, the lack of role clarity could present implementation challenges and confusion.
- Information dissemination strategy to patients with SCD needs more substance.

<u>Reviewer 5</u>:

Strengths:

- All relevant stakeholders are identified and included as full members of the research team or advisory board. Major
- A clear plan is presented that demonstrates how all stakeholders were involved in the development of the project, and will be involved in the actual project and in dissemination. Major
- National survey data demonstrates that adolescents define transition success as managing their own

disease. This data was validated in a small sample at the investigators institution. These patients also identified QOL as an important outcome. Major

Weaknesses: none noted.

Overall Comments

Reviewer 1:

Overall this is a good application. Strengths include the formative research and use of models including one developed by a key co-investigator as a basis for the development of interventions. The primary outcome, HRQL, was identified as important from a survey of people with SCD. The investigative team is highly accomplished with the necessary complementary experience and there is excellent institutional support and data and statistical infrastructure at CHOP in addition to the support from Keystone Health for a community health worker to provide the CHW intervention in Philadelphia. The major weaknesses are the limited number of patients in the transition age group, the consequence of transitioning the backlog of patients >17 years of age over 3.5 years (are there adequate numbers of adult providers to see all of these new patients?), and the optimistic estimate of the proportion of adolescents that will enroll in a clinical trial.

Reviewer 2:

Emerging adults with sickle-cell disease face higher mortality rates during the pediatric to adult transition period. Reasons for this include poor care coordination, poor patient engagement, poor self-management skills, and psychosocial issues. Lack of research in this area prevents patients from understanding what method will work best for their transition. Using the conceptual frameworks of the SMART model and the Chronic Care Model, this project aims to compare two popular programs in disease self-management and system navigation, community health workers and mobile health platforms, against a usual care method to improve quality of life and acute care. It also hopes to take a look at the effect of psychosocial factors. The project will help patients and providers determine how to effectively transition from pediatric to adult care as well as how providers can encourage self-management. There are a few moderate and minor weaknesses that should be further considered in terms of the stakeholder/researcher representation and the future of the project. Addressing these weaknesses could make the proposal stronger, but, overall, this is a very good proposal.

Reviewer 3:

Overall, this is an excellent proposal looking to compare utilization of community health workers and mobile health application interventions with enhanced standard of care, for adolescents with sickle cell disease trying to have improved transition to adult care health outcomes. The proposal clearly identifies the gap in research and knowledge about which interventions during this critical stage can help adolescents transition to adult care and minimize adverse results. It is grounded in theoretical framework and the primary outcomes such as quality of life and disease self-management have been identified as most important by the patient stakeholders.

Methodologically, study has a strong block randomized design with three arms and four repeated time points. Analytical methods are sound, justified and appropriate for the design. Research team is very diverse, inclusive of disciplines that are relevant and needed for this type of projects, as well as inclusive of various stakeholder members and groups. PIs are experienced in carrying out the projects of similar scope and duration, and their expertise compliments each other. Project is focused on closing the gap that is important to patient stakeholders (and other stakeholders as well). There is evidence of patient and stakeholder engagement in planning and designing the study, along with the plan of their involvement in study execution and dissemination.

Overall score is slightly reduced because of few minor but modifiable weaknesses. Mainly there is a discrepancy between the proposed enrollment and the sample size planned for the analysis. Also primary aims seem a bit overpowered. Finally we know that use of community health workers and mobile health applications have shown efficacy in the past studies with different outcomes, so why should we think that it

will be any different for the sickle cell disease?

Reviewer 4:

This is a 3-arm randomized, controlled trial among SCD patients assessing the efficacy of two interventions: 1. care coordination via community health workers and 2. mobile applications for improving care transitions and patient outcomes compared to usual care. The study team is a diverse composition of patients, researchers, clinicians and community stakeholders. The team is experienced in research, social services, community engagement and advocacy. The team demonstrates a strong understanding of the challenges and gaps associated with improving care transitions among SCD patients. The team has incorporated their collective experiences in addressing and defining the problem into the research design. Notably, this is demonstrated by a strong recruitment strategy which was largely derived from past experiences associated with recruitment failures. The team demonstrates an ability to retain participants in the study once enrolled.

The patient voice is strongly integrated into the study concept and design. The proposal has few weaknesses the most concerning of which is a need to better define how this information and results from this study will directly reach study participants, other patients with SCD and their social support systems and networks other than through traditional avenues for disseminating information. This study has strong implementation potential and the results may provide critical insights about which strategies may improve care transitions in SCD patients aging out of pediatrics.

From a payer perspective, while these interventions are exciting, in 2017, national policy does not fully support financial coverage for either of these interventions. While allowances have been made for reimbursement for telehealth, the restrictions around the rules governing reimbursement would not support reimbursement for app use. Medicaid programs vary in their coverage for community health workers but the field is quickly shifting toward value based purchasing and creation of strategies to reimburse providers for integrated care coordination. Both of these interventions have potential to align with this vision and studies like this can help produce evidence needed to inform policy discussions about coverage.

Reviewer 5:

This project proposes a pragmatic RCT to study differences between either a community health worker intervention or mHealth app vs. usual care in 360 adolescents young adults ages 17-22 with SCD. The primary outcomes are patient centered (QOL, self-efficacy) and health services use. Additional clinical outcomes will be measured. Sub-analysis will stratify for disease severity and differences in age (17-21 and 22 and older). Additional analysis will identify other predictors of outcomes. Outcomes will be measured at 6, 12, and 18 months after a 6 month long intervention period. A strong rationale for the need for the of transition interventions in SCD is provided citing a 7-fold increase in mortality and very large numbers of hospitalizations and ED visits. A strong rationale for the selection of the comparator interventions is provided with limited data of their use in transition with other diseases, and very limited in SCD. 450 adults for a final sample of 360 patients will be enrolled between 4 centers in PA and OH. All centers have a strong record of good recruitment and retention and are excellent centers to conduct the proposed project.

The study team is exceptionally strong with expertise in SCD, clinical trials, HSR, community work, patient and family advocates, SCD advocacy groups, payers, community advocacy groups, and policy makers. It is clear the groups were involved in the proposal development and will be very involved as members of the research team or advisory board. This strong team of collaborators has developed a very strong dissemination plan.

Enthusiasm for the proposal is weakened by a relatively simplistic description of the interventions, especially the CH worker. It is unclear how many times they will contact the subjects. It is also unclear how many will be hired and if there is sufficient budget support to hire them. Overall, this is an exceptionally strong application that proposes interventions endorsed by patients but lacking high level evidence to support widespread recommendation. A successful project could provide such evidence.

Does the application have acceptable risks and/or adequate protections for human subjects?

Reviewer 1: Yes

Reviewer 2:

Reviewer 3: Yes

Reviewer 4: Yes

Reviewer 5: Yes
